# The First Freshwater Mosasauroid (Upper Cretaceous, Hungary) and a New Clade of Basal Mosasauroids

**DOI:** 10.1371/journal.pone.0051781

**Published:** 2012-12-19

**Authors:** László Makádi, Michael W. Caldwell, Attila Ősi

**Affiliations:** 1 Department of Paleontology and Geology, Hungarian Natural History Museum, Budapest, Hungary; 2 Department of Biological Sciences, University of Alberta, Edmonton, Alberta, Canada; 3 MTA-ELTE Lendület Dinosaur Research Group, Eötvös University Department of Physical and Applied Geology, Pázmány Péter sétány 1/c, Budapest, Hungary; Ludwig-Maximilians-Universität München, Germany

## Abstract

Mosasauroids are conventionally conceived of as gigantic, obligatorily aquatic marine lizards (1000s of specimens from marine deposited rocks) with a cosmopolitan distribution in the Late Cretaceous (90–65 million years ago [mya]) oceans and seas of the world. Here we report on the fossilized remains of numerous individuals (small juveniles to large adults) of a new taxon, *Pannoniasaurus inexpectatus* gen. et sp. nov. from the Csehbánya Formation, Hungary (Santonian, Upper Cretaceous, 85.3–83.5 mya) that represent the first known mosasauroid that lived in freshwater environments. Previous to this find, only one specimen of a marine mosasauroid, cf. *Plioplatecarpus* sp., is known from non-marine rocks in Western Canada. *Pannoniasaurus inexpectatus* gen. et sp. nov. uniquely possesses a plesiomorphic pelvic anatomy, a non-mosasauroid but pontosaur-like tail osteology, possibly limbs like a terrestrial lizard, and a flattened, crocodile-like skull. Cladistic analysis reconstructs *P. inexpectatus* in a new clade of mosasauroids: (*Pannoniasaurus* (*Tethysaurus* (*Yaguarasaurus, Russellosaurus*))). *P. inexpectatus* is part of a mixed terrestrial and freshwater faunal assemblage that includes fishes, amphibians turtles, terrestrial lizards, crocodiles, pterosaurs, dinosaurs and birds.

## Introduction

Modern squamate reptiles are largely restricted to terrestrial environments with only a few species living in aquatic environments and an even smaller number, i.e., the marine iguana and sea snakes such as the acrochordids, true sea snakes and sea kraits, occupying facultative and obligatory niches in marine environments [1]. The adaptive radiation into aquatic environments by the long extinct squamate clade commonly known as mosasauroids (mosasaurs and aigialosaurs), resulted in the evolution of paddle-like limbs (hydropedality) [2] and modified hips (hydropelvia) [3], [4], a laterally compressed and downturned tail for swimming [5], modifications to the middle-ear osteology [6], [7], and a progressive increase towards gigantism within different subclades [6]. It is therefore an excellent example of a major secondarily aquatic transition in vertebrate evolution, and the only one of its kind among squamates [8]. However, despite our broad understanding of the ∼30 million year time span of mosasauroid aquatic evolution, there has never been a clearly documented example of a mosasauroid group that unequivocally occupied freshwater habitats [6], . Similar to almost all living cetaceans, all previously known mosasauroids are considered to have occupied marine habitats.

However, here we describe a new mosasauroid, *Pannoniasaurus inexpectatus* gen. et sp. nov., that inhabited freshwater environments during the Late Cretaceous of Hungary, similar to the ecology of modern freshwater river dolphins (Amazon, Ganges, Yangtze, La Plata Rivers) [12].

### Locality

**Figure 1:**
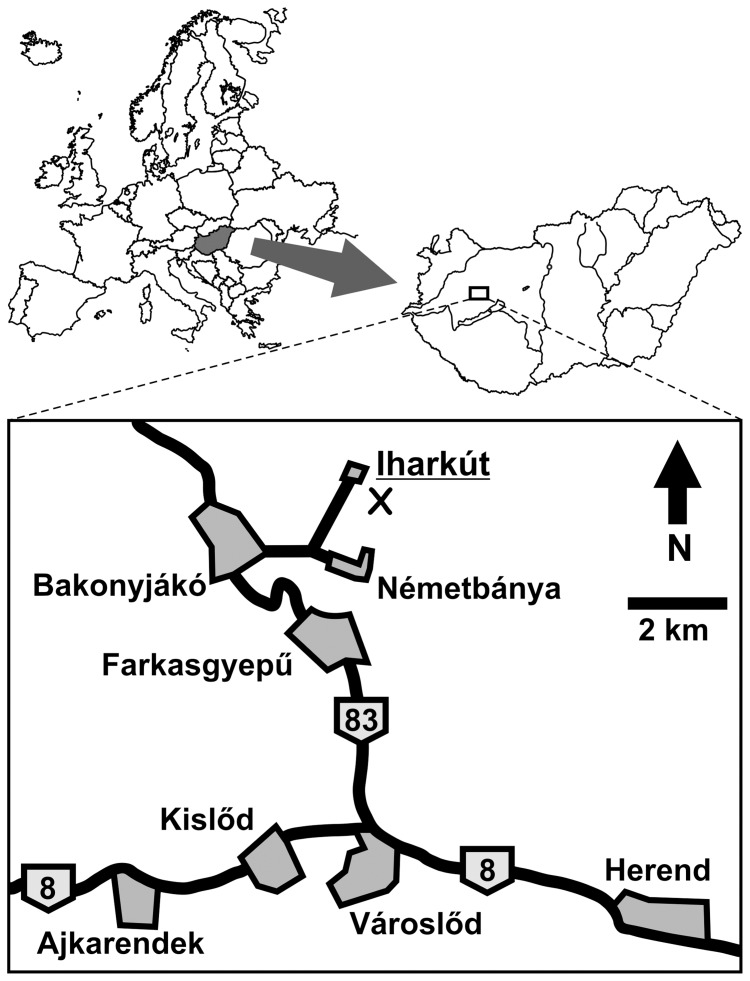
Location map of the Iharkút locality in Hungary. “X” marks the locality.

The Iharkút fossil vertebrate locality (referred to as Iharkút in memory of the village of Iharkút that was destroyed in order to create the mine) that yielded the numerous remains of *Pannoniasaurus* is located in an open-pit bauxite mine near Németbánya, Bakony Mountains, Western Hungary (Figure 1). The mine exposes the base of the Csehbánya Formation (Santonian, Upper Cretaceous), an alluvial floodplain deposit that contains various vertebrate remains, as well as invertebrate and plant fossils. To date, the documented vertebrate fauna includes lepisosteid and pycnodontiform fishes [13], albanerpetontid and anuran amphibians [14], bothremydid turtles [15], lizards [16], alligatoroid, ziphosuchian and heterodont eusuchian crocodiles [13], [17], azhdarchid pterosaurs [18], a rhabdodontid ornithopod [19], the ceratopsian dinosaur *Ajkaceratops* [20], the basal nodosaurid ankylosaur *Hungarosaurus* [21], [22], theropods [23], and enantiornithine birds [24].

In the Late Cretaceous the Iharkút area where *P. inexpectatus* lived was part of an alluvial floodplain system on an island landmass in the western Tethyan archipelago. Besides vertebrates and freshwater invertebrates, plant fossils were also unearthed and provide evidence that the area was covered by terrestrial vegetation [13], [25].

### Geology of the Locality

**Figure 2:**
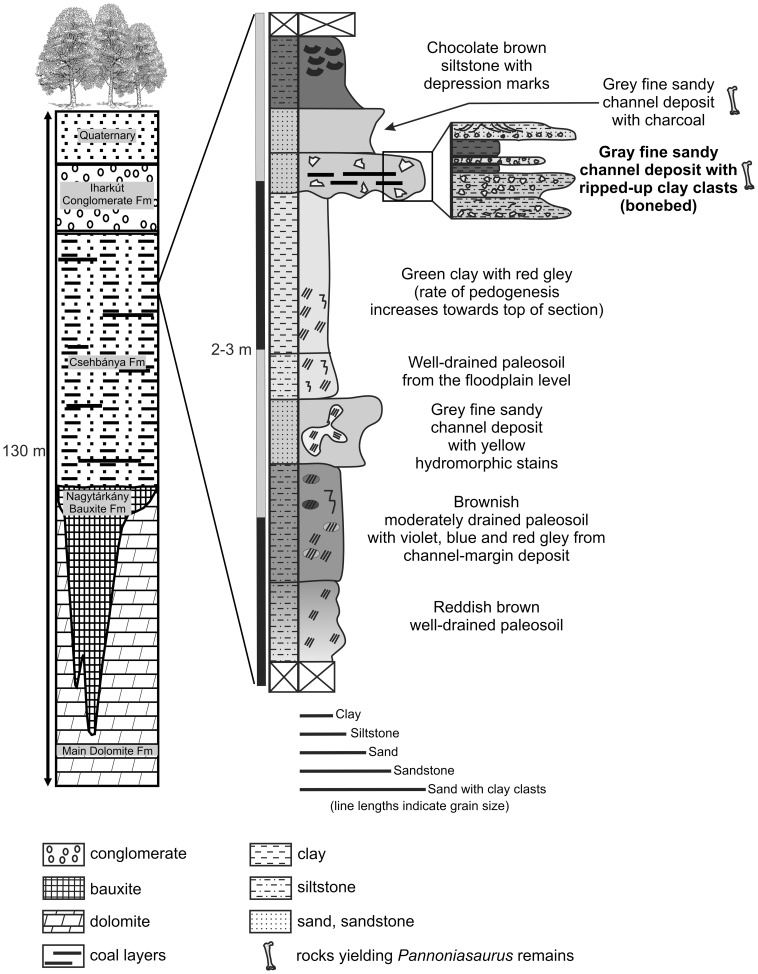
Geology of the Iharkút locality. Hypothetical geological section and detailed partial stratigraphic column at the most important SZ-6 site.

The bone-yielding Csehbánya Formation (Figure 2) overlies the Nagytárkány Bauxite Formation and the Triassic Main Dolomite Formation. It is a floodplain and channel deposit built up of variegated clay, silt with interbedded grey and brown sand, sand and sandstone beds, as well as paleosoils. In some exposures of the open-pit mine area, the Csehbánya Formation is sometimes covered by the Eocene Iharkút Conglomerate Formation; in other locations it is covered by the Oligocene–Miocene Csatka Formation or sits immediately below Quaternary deposits [25]–[29]. Detailed palynological studies suggest a Santonian age for the Csehbánya Formation [28], a date that is also supported by recent paleomagnetic data [13], [25].

At Iharkút, vertebrate remains, including those of *Pannoniasaurus inexpectatus* gen. et sp. nov., were found throughout exposures of the Csehbánya Formation, but the SZ-6 site is without question the most important site. This outcrop is an approximately 2–3 m thick sequence of beds built up of coarse, pebbly sand and organic-rich silt and clay, which are interpreted as crevasse splay deposits (Figure 2). The base of the sequence is clearly erosional as it forms noticeable erosional surfaces into the floodplain deposits. The bonebed at SZ-6 is a 10 to 50 cm thick, basal breccia composed of gray sand, siltstone, clay clasts, pebbles, and plant debris (also charcoal) that occasionally contains surprisingly complete bones, but more frequently yields fragmentary bones; the basal breccia is sometimes interrupted by finer sediments that settled out under calmer circumstances [13]. As a result of these alternating energy conditions of deposition, bones in different states of preservation can be found in the same bed. Nearly 80% of the vertebrate remains from Iharkút were discovered in the bonebed of this site [13], [25].

The sandstone bed covering the basal breccia also contains vertebrate fossils, but the bones are fewer in number and more poorly preserved. However, two incomplete associated skeletons of the nodosaurid ankylosaur *Hungarosaurus tormai* have been found in this bed. The overlying bed is a laminated, grayish siltstone of variable thickness (30 cm to 1.5 m) and contains plant debris and only a few bones, but this bed also yielded two partial associated skeletons of *Hungarosaurus*. The sequence is closed by a greyish siltstone of several meters thickness in which vertebrate remains are extremely rare [13], [25].

It is worth mentioning that several similar sequences are exposed within the mine. In most of these cases the basal breccia (bonebed) is missing and the cycle starts with sandstone, or, if present, the basal breccia is thin (only a few centimeters, containing no, or only a few vertebrate remains). Moreover, the cycles sometimes end with paleosoils, which also might contain vertebrate remains, mostly dinosaur and crocodile teeth, and bone and turtle shell fragments [13], [25].

## Materials and Methods

No permits were required for the described study, which complied with all relevant regulations.

### Material

The holotype and referred specimens have been collected from the alluvial sediments of the Csehbánya Formation from various exposures at the Iharkút open-pit bauxite mine, Bakony Hills, Western Hungary since the discovery of the locality in 2000. A single vertebra has been collected in 1999 from the interdigitating Ajka Coal Formation at the waste dump of the coal mines next to the town of Ajka, 20 kms from Iharkút [13], [28], [29]. Currently more than one hundred bones of *Pannoniasaurus* (See Appendix S1), sourced from a large number of individuals of differing age classes, are known from the alluvial flood-plain [30] deposits that comprise the Csehbánya Formation. All specimens of *Pannoniasaurus* are housed in the Hungarian Natural History Museum (Magyar Természettudományi Múzeum: MTM), Budapest, Hungary.

Though all remains (including the holotype) of *Pannoniasaurus* are isolated bones, the density of the specimens, the various size classes, the large number of similar elements from individual animals, and their unique characters, make it possible to link them together into a single taxon.

For example, the logic we have applied in identifying a single taxon of mosasauroid from the Iharkút assemblage is as follows: if in an assemblage of extant lizard vertebrae one finds that all the vertebrae show a similar diagnostic feature (e.g., in extant *Varanus* all vertebrae display a flared condyle), then it is reasonable to conclude that all of the lizards represented are morphologically *Varanus* (a morphospecies concept is being applied); it would therefore logically follow that if there is one vertebra that differs, then the application of a morphospecies concept and its criterion for inclusion/exclusion would be that there are at least two morphotaxa present, not one.

Therefore, as is the case for the Iharkút fauna, and staying with the vertebral example given above, if all of the presacral vertebrae assigned to *Pannoniasaurus* possess a common characteristic (in this case there are many) that is shared regardless of size class or presumed position in the vertebral column, then it is reasonable to conclude there is only a single morphotaxon present in the fauna. The case for only a single aigialosaur-grade mosasauroid morphotaxon being present in the Iharkút assemblage is reinforced by the application of this same methodology to the multiple specimens of the same skull, lower jaw, and appendicular skeletal elements regardless of size. In the final analysis, there simply is no evidence of more than one morphotaxon of mosasauroid, nor of any other large-sized squamate, at the locality. Similar methodologies have been used by other authors, e.g. Houssaye et al. [31] referred newly discovered cervical vertebrate to *Pachyvaranus crassispondylus*, though the species was originally described on the basis of dorsal vertebrae.

### Phylogenetic Methods

Phylogenetic analysis of thirty-two taxa and one hundred and thirty five characters was conducted using a taxon-character matrix from a recent cladistic analysis of mosasauroid squamates [4]. That character list and taxon-character matrix originates from a previous work [32], with characters deleted/added/modified through later studies [2], [3]. In order to make it easier for the reader to follow the changes made through that series of studied, the character list (See Appendix S2) was recompiled by quoting almost word-by-word from the original character list of Bell [32] and including all changes made by later studies [3], [4].

The data matrix, available online (See Appendix S3), was modified with the recoding of *Tethysaurus* (characters 1, 87, 88, 98, 105, 107 and 128) based on personal observation and the original description [33] and with the addition of new character data from *Pannoniasaurus.*

The analysis was performed using PAUP version 4.0b10 [34]. All multistate characters were unordered and unweighted. The data matrix was analyzed using heuristic search algorithms.

### Nomenclatural Acts

The electronic edition of this article conforms to the requirements of the amended International Code of Zoological Nomenclature, and hence the new names contained herein are available under that Code from the electronic edition of this article. This published work and the nomenclatural acts it contains have been registered in ZooBank, the online registration system for the ICZN. The ZooBank LSIDs (Life Science Identifiers) can be resolved and the associated information viewed through any standard web browser by appending the LSID to the prefix "http://zoobank.org/". The LSID for this publication is: urn:lsid:zoobank.org:pub:29161BAD-A892-46F0-AB28-1EE594A229A1. The electronic edition of this work was published in a journal with an ISSN, and has been archived and is available from the following digital repositories: PubMed Central, LOCKSS.

## Results

### Systematic Paleontology

Squamata Oppel, 1811 [35].

Mosasauroidea Camp, 1923 [36].

Familia incertae sedis.

Tethysaurinae subfam. nov.

urn:lsid:zoobank.org:act:A71608F3-EB46-4B25-89E2-050CD0A84EA1.

#### Definition (node-based)

The most recent common ancestor of *Pannoniasaurus inexpectatus* and *Russellosaurus coheni* Polcyn & Bell, 2005 [37] and all its descendants.

#### Type genus

*Tethysaurus* Bardet, Pereda-Suberbiola & Jalil, 2003 [33].

#### Composition

*Pannoniasaurus inexpectatus* gen. et sp. nov., *Tethysaurus nopcsai* Bardet, Pereda-Suberbiola & Jalil, 2003 [33], *Yaguarasaurus columbianus* Páramo, 1994 [38] and *Russellosaurus coheni* Polcyn & Bell, 2005 [37].

#### Diagnosis

Medium-sized (max. 6 meters) mosasauroids exhibiting combination of primitive characters: predental rostrum absent; premaxilla-maxilla suture ends anterior to or level with the midline of the fourth maxillary tooth; nearly straight frontoparietal suture; quadrate alar concavity shallow; elongated stapedial pit (at least three times longer than wide); quadrate distal condyle saddle-shaped, upward deflection of quadrate distal condyle absent; mandibular glenoid formed mainly by articular; cervical synapophyses extend below ventral border of centrum; dorsoventrally compressed centra in precaudal vertebrae; two sacrals with large ribs/transverse processes subcircular/oval in cross-section; facet for ilium on tip of sacral transverse processes; very elongated (two times longer than wide) pontosaur-like caudal centra; anteroposteriorly narrow scapula; ilium with posterior iliac process with compressed dorsal end bearing longitudinal grooves and ridges, and spoon-shaped preacetabular process overlapping the pubis.

#### Comments

*Tethysaurus* was chosen as type genus because it is the best-represented genus of the subfamily, known from multiple partial skeletons. Thus the subfamily name derives from the name of its type genus.

*Pannoniasaurus* gen. nov.

urn:lsid:zoobank.org:act:503B06AC-91D0-4D6B-B365-FBA9A4EBEB6E.

*Pannoniasaurus inexpectatus* sp. nov.

urn:lsid:zoobank.org:act:3DD4ED72-9F20-4D87-85B6-2C1D81DBA6E8.

2006 Mosasauridae incertae sedis, Makádi et al., p. 497 [39].

2009 Mosasauridae, Kocsis et al., p. 1 [40].

2012 Mosasauroidea indet., Ősi et al., p. 549 [13].

#### Holotype

**Figure 3:**
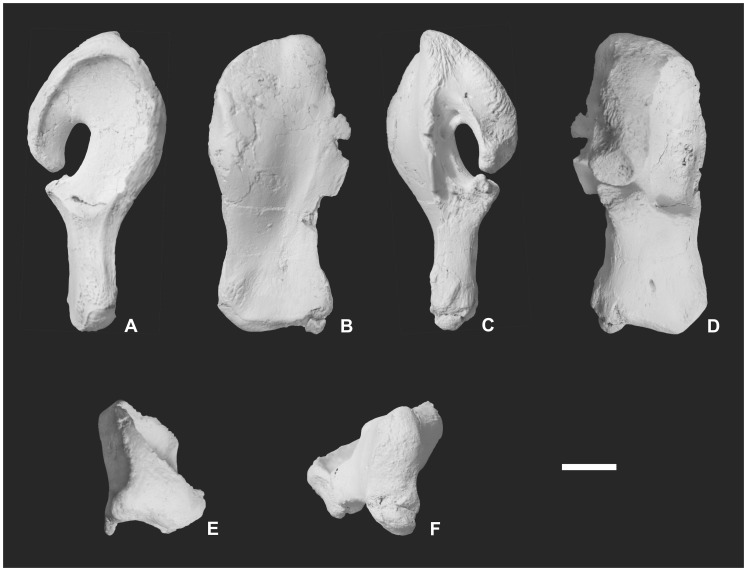
Holotype *of Pannoniasaurus inexpectatus*. Quadrate (MTM 2011.43.1.) in lateral (A), anterior (B), medial (C), posterior (D), dorsal (E) and ventral (F) views. Scale bar represents 1 cm.

**Figure 4:**
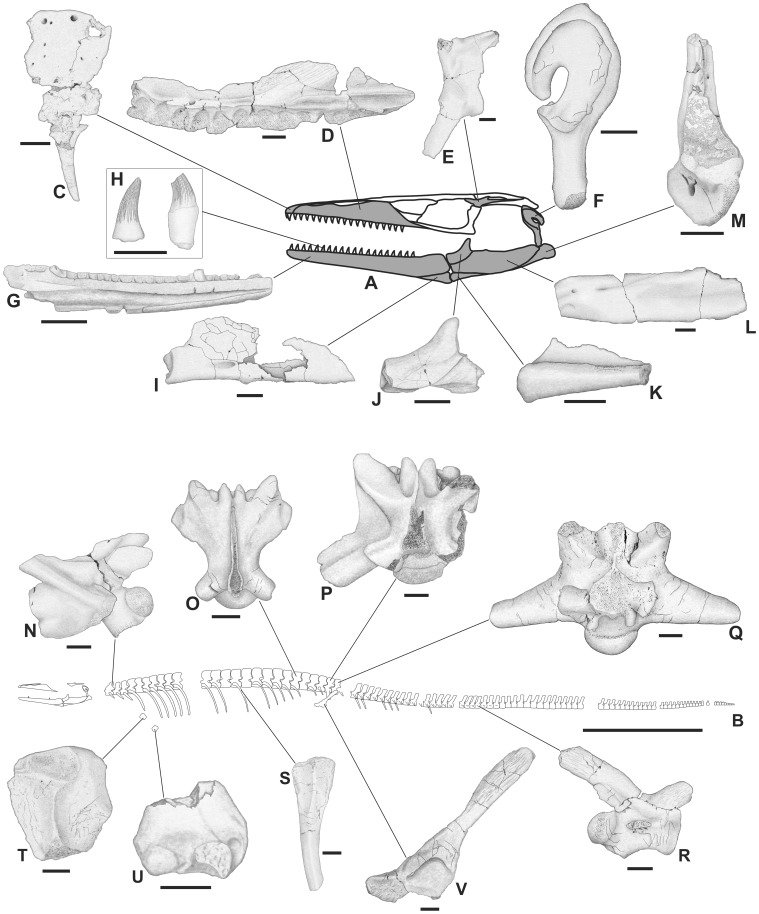
Skeletal anatomy of *Pannoniasaurus inexpectatus*. A: skull drawing shows known elements in grey. B: skeletal reconstruction showing preserved bones in white. C: premaxilla (MTM 2007.25.1.) in dorsal view. D: right maxilla (MTM 2007.29.1.) in lingual view. E: left postorbitofrontal (MTM 2007.28.1.) in dorsal view. F: right quadrate (MTM 2011.43.1.) in lateral view. G: left dentary (MTM 2007.37.1.) in lingual view. H: isolated teeth without and with base preserved. I: left splenial (MTM 2011.41.1.) in lingual view. J: right coronoid (MTM 2007.23.1.) in lingual view. K: left angular (MTM 2007.36.1.) in labial view. L: right surangular (MTM 2007.30.1.) in labial view. M: left articular (MTM 2007.39.1.) in dorsal view. N: mid-cervical vertebra (MTM V.01.149.) in lateral view. O: dorsal vertebra (MTM V.01.222.) in dorsal view. P: first sacral vertebra (MTM Gyn/122.) in dorsal view. Q: second sacral vertebra (MTM Gyn/121.) in dorsal view. R: anterior caudal vertebra (MTM Gyn/104.) in lateral view. S: rib fragment (MTM 2007.89.1.) in dorsolateral view. T: proximal end of left humerus (MTM 2007.42.1.) in flexor view. U: distal end of right humerus (MTM 2011.42.1.) in flexor view. V: left ilium (MTM 2007.40.1) in lateral view. All known elements of *Pannoniasaurus* are isolated bones from multiple individuals and many are known as multiple specimens but only one is figured. Vertebrae and ribs not figured in (B) symbolize that no complete series has been found. Scale bars represent 1 cm (A, C-V) and 1 meter (B).

MTM 2011.43.1., isolated right quadrate (Figures 3, 4F).

#### Paratypes

MTM V.01.115., left quadrate; MTM 2007.31.1., fragmentary left quadrate.

#### Referred specimens

2 isolated premaxillae; 3 maxillae; 2 postorbitofrontals; 2 quadrates; 3 dentaries; 3 splenials; 3 angulars; coronoid; 2 surangulars; articular; 91 isolated teeth; 20 cervical, 40 dorsal, 4 sacral, and 18 caudal vertebrae; 34 vertebral fragments; 3 ribs; 2 humeral fragments; 4 ilia (See Appendix S1 for inventory numbers). Since all remains are isolated bones, the basis for the referral of this material to *Pannoniasaurus* is explained above in the “Materials and Methods” section.

#### Specific diagnosis (as for genus by monotypy)

Medium-sized (max. 6 meters) mosasauroid with following combination of characters: quadrate stapedial pit length/width ratio of 3∶1; saddle-shaped mandibular condyle of quadrate; quadrate shaft, distal to the conch and infrastapedial process, long and gracile; large infrastapedial process; long suprastapedial process with tip angled medially; dorsoventrally flattened, laterally wide, violin-shaped premaxilla in dorsal view; high dorsal process on coronoid; teeth with carinae and anastomosing longitudinal striae; hypapophyseal peduncles with circular articulation surface; large ventrolateral crests on cervicals; parazygosphenal and paracotylar foramina absent; well-developed precondylar constriction on precaudal vertebrae; ribs with oval heads.

#### Locality, horizon and age

Csehbánya Formation, Iharkút open-pit bauxite mine, Bakony Hills, Western Hungary and interdigitating Ajka Coal Formation, Ajka coal mines, Bakony Hills, Western Hungary. Both formations are dated to the Santonian (85.8–83.5 mya), Upper Cretaceous [13], [28], [29].

#### Etymology

The generic name is derived from the ancient Roman province “*Pannonia*” in the Transdanubian part of Hungary and “*saurus*”, New Latin word from Greek ‘*sauros*’, meaning lizard; the specific epithet “*inexpectatu*s”, meaning unexpected in Latin, refers to the unexpected occurrence of this mosasaur in freshwater environments.

### Osteological Description

**Figure 5:**
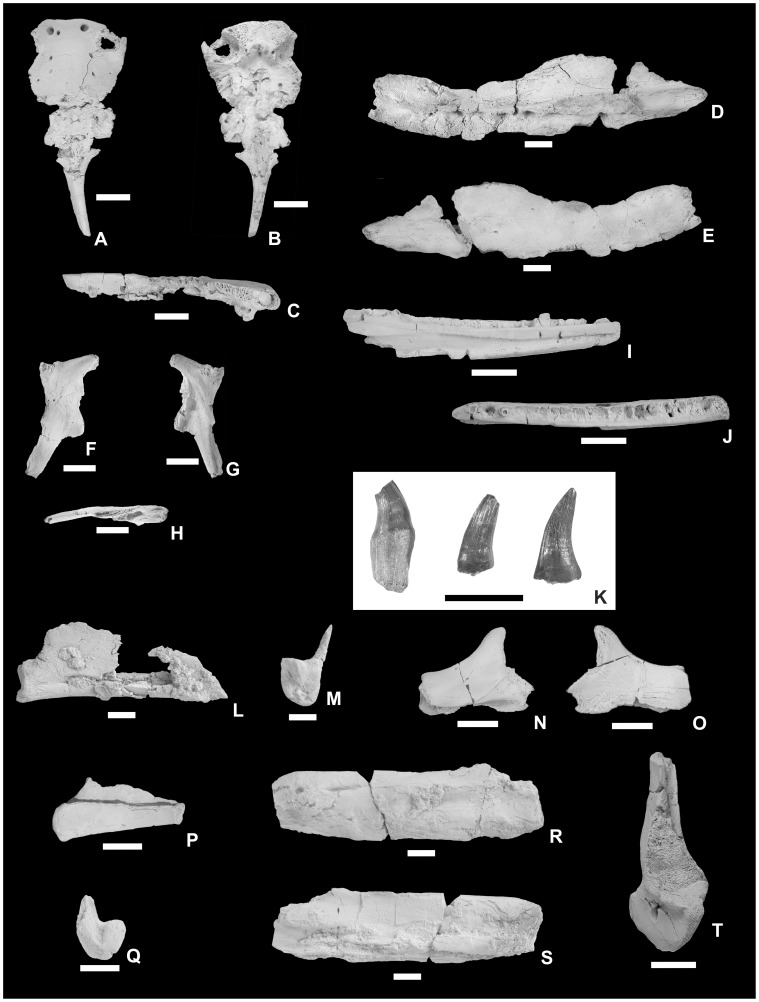
Skull and lower jaw elements, and teeth of *Pannoniasaurus inexpectatus*. Premaxilla (MTM 2007.25.1.) in dorsal (A), ventral (B), and right lateral (C) views. Right maxilla (MTM 2007.29.1.) in lingual (D) and labial (E) views. Left postorbitofrontal (MTM 2007.28.1.) in dorsal (F), ventral (G), and medial (H) views. Left dentary (MTM 2007.37.1.) in lingual (I) and occlusal (J) views. Isolated teeth isolated teeth without and with base preserved (K). Left splenial (MTM 2011.41.1.) in medial (L) and posterior (M) views. Left coronoid (MTM 2007.23.1.) in lateral (N) and medial (O) views. Right angular (MTM 2007.36.1.) in lateral (P) and anterior (Q) views. Right surangular (MTM 2007.30.1.) in lateral (R) and medial (S) views. Right articular (MTM 2007.39.1.) in dorsal view. Scale bars represent 1 cm.

The premaxilla (Figures 4C, 5A−C) is broadly arcuate, dorsoventrally flattened, and violin-shaped in dorsal view. The maxilla (Figures 4D, 5D, 5E) is also flattened by the inclination of the anterodorsal structures towards the midline. The preserved part of the maxilla bears 12 tooth sockets but the original maxillary tooth count might have been much higher. On the smaller postorbitofrontal a suture between postfrontal and postorbital is visible. The quadrate (Figures 3, 4F) is characterized by an extremely elongate shaft between the ventral condyle and the infrastapedial process (the latter being more developed than in *Russellosaurus* [37], *Tethysaurus* [33], and *Yaguarasaurus* [38], [41]). As a result, the quadrate conch is half the total height of the quadrate. The stapedial pit is three times longer than wide. The suprastapedial process is longer than in *Tethysaurus* [33], slender and does not contact the infrastapedial process. The distal condyle is more saddle-shaped than in *Tethysaurus* [33]. The dentary (Figures 4G, 5I, 5J) has a medial parapet half the height of the lateral one but bears at least 20 tightly fitted distinct alveoli. The dorsomedial process of the splenial (Figures 4I, 5L, 5M) seems to be as developed as in aigialosaurs and *Tethysaurus* [33]. The coronoid (Figures 4J, 5N, 5O) has an extremely high dorsal process as in terrestrial varanoids [42]. The angular (Figures 4K, 5P, 5Q) face is nearly circular. The retroarticular process of the articular (Figures 4M, 5T) is angled at approximately 45° and has a single large foramen on its medial surface [6], [43]. The mandibular cotyle is formed mainly by the articular, similar to *Tethysaurus* [33].

We also attribute to *Pannoniasaurus* a number of isolated teeth (Figures 4H, 5K) that are similar to *Halisaurus*, i.e., conical and curved posterolingually, bear crowns with fine anastomosing longitudinal striae, and a strong mesial but weaker labiodistal carina [44], [45].

**Figure 6:**
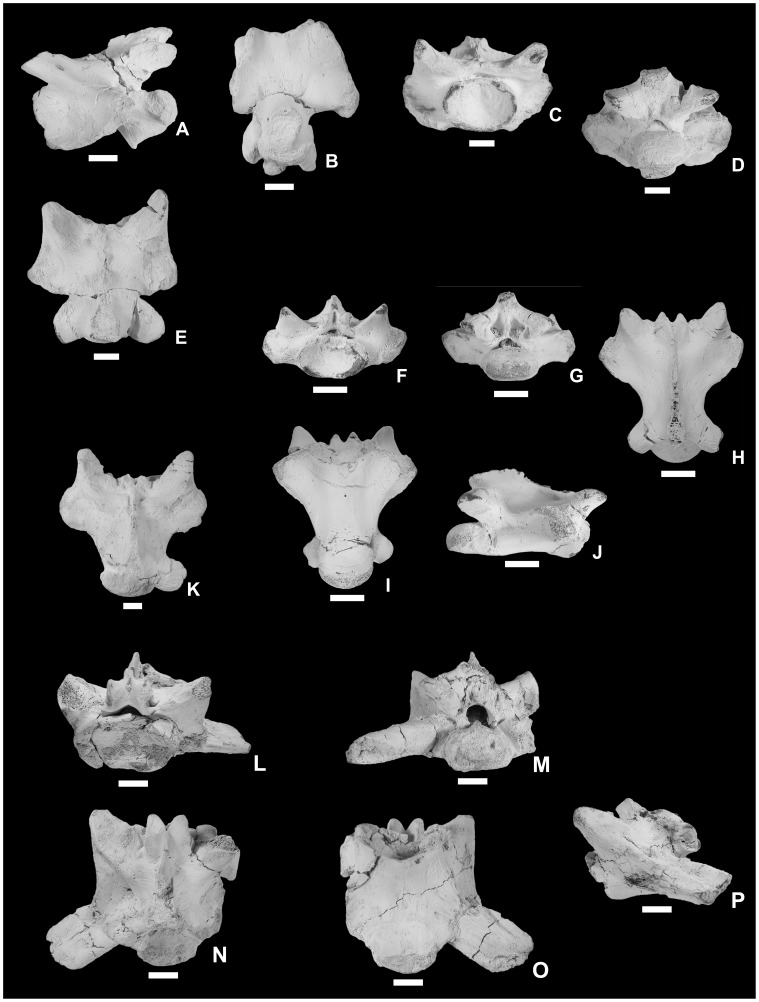
Cervical and dorsal vertebrae and first sacral vertebra of *Pannoniasaurus inexpectatus*. Mid-cervical vertebra (MTM V.01.149.) in left lateral (A), and ventral (B) views. Posterior cervical vertebra (MTM V.2000.19.) in anterior (C), posterior (D), and dorsal (E) views. Dorsal vertebra (MTM V.01.222.) in anterior (F), posterior (G), dorsal (H), ventral (I), and right lateral (J) views. Dorsal vertebra (MTM Gyn/114.) exhibiting precondylar constriction in dorsal (K) view. first sacral vertebra (MTM Gyn/122.) in anterior (L), posterior (M), dorsal (N), ventral (O), and left lateral (P) views. Scale bars represent 1 cm.

**Figure 7:**
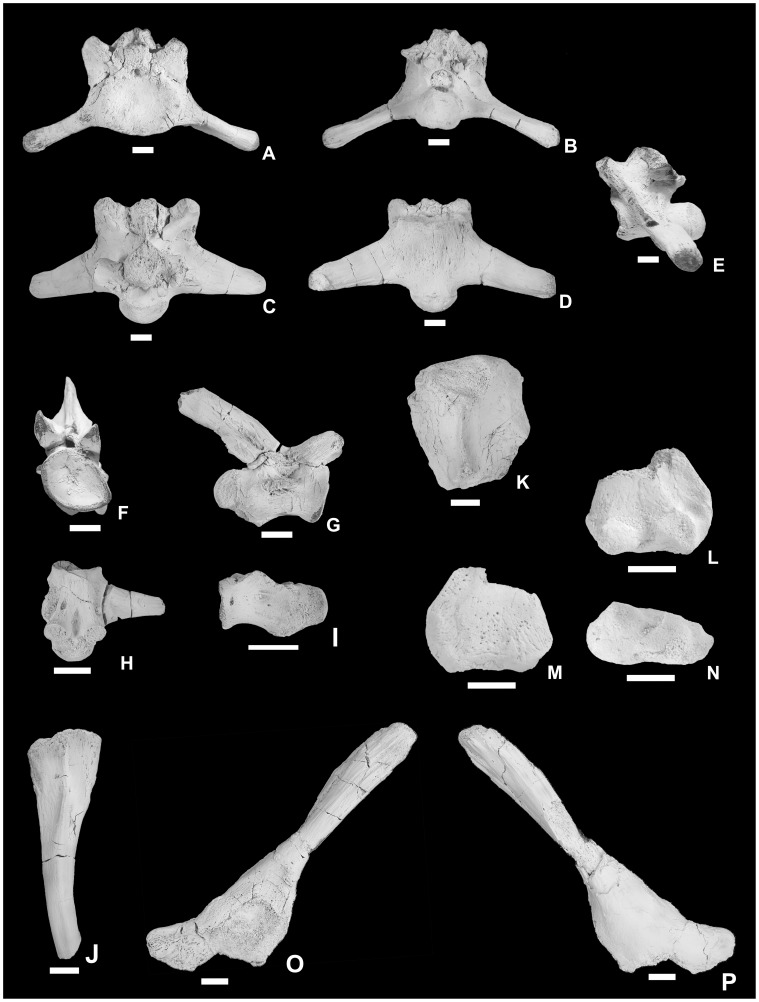
Second sacral vertebra, caudal vertebrae, rib, humerus, and ilium of *Pannoniasaurus inexpectatus*. second sacral vertebra (MTM Gyn/121.) in anterior (A), posterior (B), dorsal (C), ventral (D), and left lateral (E) views. Anterior caudal vertebra (MTM Gyn/104.) in anterior (F), and right lateral (G) views. Anterior caudal vertebra (MTM 2007.46.1.) in ventral view (H). Posterior caudal vertebra (MTM 2007.99.1.) in left lateral view (I). Left rib fragment (MTM 2007.89.1.) in lateral view (J). Proximal end of left humerus (MTM 2007.42.1.) in flexor view (K). Distal end of right humerus (MTM 2011.42.1.) in flexor (L), extensor (M) and distal (N) views. Left ilium (MTM 2007.40.1) in lateral (O) and medial (P) views. Scale bars represent 1 cm.

The vertebrae (Figures 4N−R, 6A−P, 7A−I) of *Pannoniasaurus* are similar in almost all respects (size, shape, size and shape of processes, and intracolumnar variation) to those of *Tethysaurus* [33], and cannot be compared to either *Russellosaurus* [37] or *Yaguarasaurus* [38], [41] as postcranial remains are almost entirely absent for both taxa. In all presacral vertebrae of *Pannoniasaurus inexpectatus* the condyles/cotyles are oval and oblique, and the vertebral condyles are flared ( = precondylar constriction) (most pronounced on juvenile cervicals), in contrast to all known mosasauroids [6], [43], but similar to varanids [46]. The cervicals (Figures 4N, 6A−E) have compressed centra similar to *Tethysaurus* and *Halisaurus* [33], [45], [47], and bear small zygosphenes and zygantra. Large crests extend between the synapophyses and anterior edge of the cotyles and project ventrally below the ventral surface of the centrum as in *Halisaurus* [47], [48] and *Tethysaurus* [33], and are morphologically similar to the dorsally positioned pterosphenes of palaeophiid snakes [49]. Hypapophyseal peduncles have circular articulation surfaces in contrast to *Tethysaurus* [33]. Zygosphenes/zygantra are small on the anteriormost dorsals, similar to the cervicals. Further posteriorly on the dorsals (Figures 4O, 6F−K) the zygosphenes/zygantra become large and functional. Contrary to the condition in *Tethysaurus*, no vertebrae exhibit parazygosphenal and paracotylar foramina [33]. In contrast to derived mosasauroids, i.e., taxa within the grade of hydropelvic mosasaurs [6], [43], there appear to be two sacrals. The massive first sacral (Figures 4P, 6L−P) is short and broad. The posteroventrally projecting transverse processes are crescent-shaped in cross-section and share a broad platform with the prezygapophyses. The zygosphenes of the first sacral are the largest in the vertebral series. The second sacral (Figures 4Q, 7A−E) is similar to the first in general shape but its centrum and transverse processes are less robust, the latter being subcircular in cross-section. The distal end of the transverse process bears a facet for the articulation with the ilium. Zygosphenes/zygantra are present. The caudals (Figures 4R, 7F−I) are similar to those of *Tethysaurus* [33] and to pontosaurs [50]. The centra are elongate, being app. two times longer than wide (width/length ratio: 1.8–2.3) with the anterior caudals being slightly shorter compared to the posterior ones. The condyles/cotyles are circular, the posteriorly directed haemapophyses are large, the haemal arches are unfused, the prezygapophyses are elongate, while the postzygapophyses are small on the neural spines, and zygosphenes/zygantra are absent. The anterior caudals bear well-developed transverse processes. The neural spines are nearly two times the length of the centra and project posteriorly. The ribs (Figures 4S, 7J) have a shape typical of mosasaurs [6], [43] and their heads heads are more oval than in *Tethysaurus* (LM, personal observation).

The proximal humeral epiphysis (Figures 4T, 7K), though partial, appears to be part of a much longer element, similar to the expected condition for a plesiopedal limb [4]. It is mediolaterally compressed and the deltopectoral crest is undivided. Similarly, an isolated right distal tip (Figures 4U, 7L−N) shows a well-fused diaphysis, with well-developed ent- and ectepicondyles and an ectepicondylar groove. The ilium (Figures 4V, 7O−P) is robust with a long posterior blade, a well-developed acetabular face, a well-developed ventral facet for the ischium and a spoon-shaped preacetabular process that overlapped the pubis. This process is similar to that seen in many extant lizards and the mosasauroids *Aigialosaurus dalmaticus* [3] and *Tethysaurus nopcsai* (LM, personal observation) and represents the plesiopelvic condition [3], [4].

### Results of the Phylogenetic Analysis

**Figure 8:**
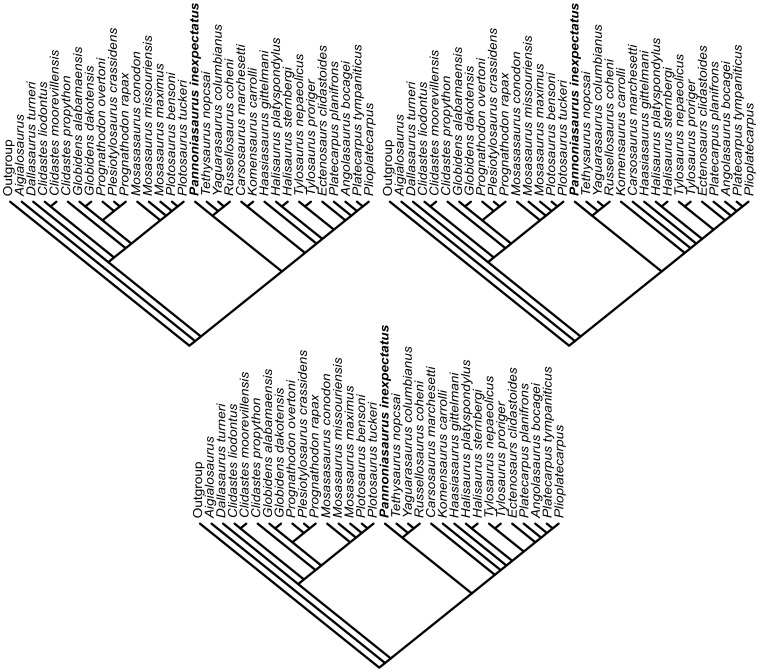
Three most parsimonious trees from phylogenetic analysis. Length  = 374, CI  = 0.4679, RI  = 0.7375, HI  = 0.5508; using 135 morphological characters and 32 taxa of mosasauroids.

**Figure 9:**
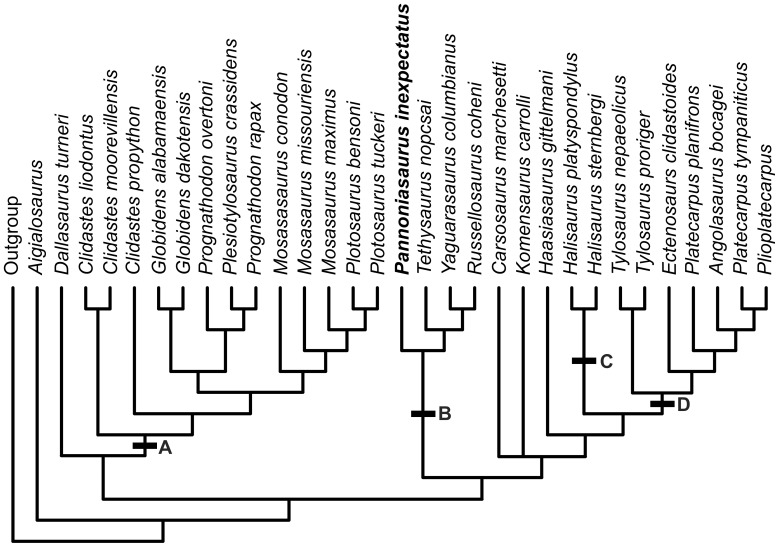
Strict consensus tree from phylogenetic analysis. Consensus tree from three most parsimonious trees (length  = 374, CI  = 0.4679, RI  = 0.7375, HI  = 0.5508) based on taxon-character matrix of 135 morphological characters and 32 taxa of mosasauroids. A: Mosasaurinae. B: Tethysaurinae. C: Halisaurinae. D: Tylosaurinae+Plioplatecarpinae.

Our analysis found three equally most parsimonious trees (length  = 374, CI  = 0.4679, RI  = 0.7375, HI  = 0.5508) that place *Pannoniasaurus* in a new clade, the Tethysaurinae. The three trees (Figure 8) reconstruct *Pannoniasaurus* as the sister taxon to the clade that includes *Tethysaurus nopcsai* (Early Turonian), *Yaguarasaurus columbianus* (?Late Turonian) and *Russellosaurus coheni* (Middle Turonian). This clade was recognized previously as a monophyletic group [2], [4], but not in the sister group position found in this analysis (Figure 9).

The Tethysaurinae (*Pannoniasaurus* (*Tethysaurus* (*Yaguarasaurus*, *Russellosaurus*))) (Figure 9B) is reconstructed at the base of a clade that includes the aigialosaurs *Carsosaurus*, *Komensaurus*, and *Haasiasaurus*, and the clades that include conventional marine mosasaur-grade mosasauroids such as halisaurs, tylosaurs and plioplatecarpines. The concept of a monophyletic clade of aigialosaurs from within which a polyphyletic Mosasauridae arises (two major lineages of a grade of paddle-bearing marine mosasaurs) [11], is supported here, and provides further support for the hypothesis of convergent aquatic adaptations in paddle-bearing mosasaurs [2−4], .

## Discussion

### Comments on the *Pannoniasaurus* Material, its Taphonomy and Geochemical Analysis

Since the SZ-6 bonebed (Figure 2), which yielded most *Pannoniasaurus* remains, is depositionally a crevasse splay and was formed in a short time interval, likely as a result of a flood, non-weathered and unabraded vertebrate remains preserved in this bonebed originate from animals that might have died shortly before final burial [13], [25], [51]. Considering the tropical climate, bones lacking signs of weathering might not have been exposed or were temporarily buried before they were picked up by the flood and deposited in the bonebed. Bones lacking abrasion, as well as breakage angles, suggest rapid burial, showing that they were not transported very far, which indicates that the animals lived and died close to the place of accumulation [13].

It is important to note that a single vertebra of *Pannoniasaurus* (MTM V.2000.21.), as well as a variety of fish and crocodile teeth, were collected from the waste dump of the subterranean Ajka coal mine. The Ajka Coal Formation interdigitates with the Csehbánya Formation, the depositional environment of the latter was a floodplain, while the Ajka Coal Formation was formed in the accumulation basin of the same river system [30]. Both of these facies were formed in the same paleogeographic area, which itself might have been part of a larger, but isolated landmass, as suggested by endemic taxa such as *Iharkutosuchus* [17], *Ajkaceratops* [20], *Hungarosaurus* [21], [22], or *Pannoniasaurus* itself.

Throughout the scientific study of the Csehbánya and Ajka Coal Formations (lasting back more than a century), these sediments are known to contain terrestrial plant and freshwater invertebrate fossils, the former yielding the continental vertebrate fauna discovered in 2000 [13], [29]. The Csehbánya Formation has never produced a single marine or brackish faunal or floral element. Besides lithological and sedimentological evidence, floral (i.e. leaf imprints and carbonized tree trunks) and faunal elements (i.e. freshwater invertebrates, freshwater and terrestrial vertebrates) also suggest that the environment of deposition was a freshwater river system.

In the Ajka coal mines area, where the waste dump of the mine yielded the single *Pannoniasaurus* vertebra, only the upper part of the Ajka Coal Formation is known to contain marine invertebrates as transgression proceeded. The lower part of the formation contains freshwater molluscs and ostracods [25], [52]. The piece of rock that yielded the single vertebra is typical of the lower part of the formation, and contains abundant freshwater molluscs. Thus there is no evidence of *Pannoniasaurus* occurring outside freshwater environments.

As with the other vertebrate remains found in the Iharkút assemblage, bones and teeth of *Pannoniasaurus* are known from multiple horizons of the Csehbánya Formation in the mine (though most of its remains were excavated from the SZ-6 bonebed). The single *Pannoniasaurus* vertebra from the Ajka Coal Formation, though compressed, is clearly not transported and indicates the presence of *Pannoniasaurus* also in that area. These facts demonstrate that *Pannoniasaurus* was abundant spatially and temporally within the Iharkút paleogeographic unit.

The size of individual bones of *Pannoniasaurus*, when compared to those of various mosasaurs [6] and large bodied extant lizards, indicates that the total length of the largest specimens was about 6 meters, though most bones appear to refer to body lengths of approximately 3–4 meters; the smallest vertebrae suggest individuals with a total length of about 70 cm. Taphonomical studies of the vertebrate material of the locality suggest that there was no filter effect by size in the accumulation process [13], thus this size range of *Pannoniasaurus* remains seems to represent the population correctly. These evidences suggest that many individuals of various size and age classes of *Pannoniasaurus* were present in the area over a short time interval, suggesting that an entire population of *Pannoniasaurus* was living in these river systems as opposed to migrating into them for seasonal food opportunities or reproduction.

A recent geochemical study [40] investigated the taphonomical and ecological differences among different vertebrates found at Iharkút. This study has proven that all the fossils belonging to different taxonomic groups from Iharkút have identical, generally high Rare Earth Element patterns as a result of early diagenetic maturation or recrystallization of the fossils, and identical Nd isotope compositions, which suggest a common diagenetic alteration history. Thus, the common presence of different taxa at the Iharkút site is unlikely to be related to reworking from different sedimentary units. Moreover, the δ^18^O_PO4_ values of *Pannoniasaurus* (and fish of the group Pycnodontiformes) are most compatible with a freshwater paleoenvironment, and are incompatible with these species having lived in Cretaceous seawater. δ^18^O_CO3_ values in all fossils from Iharkút were homogenized by diagenesis, thus ecological implications could be made only from the oxygen isotope composition of the phosphate. Finally, the ^87^Sr/^86^Sr ratios both of *Pannoniasaurus* and the Pycnodontiformes are inconsistent with a marine paleohabitat for these taxa. Thus the geochemical and isotopic data are most compatible with *Pannoniasaurus* having lived in a predominantly freshwater ecosystem [40].

Such evidence suggests strongly that *Pannoniasaurus* was not a seasonal migrant from marine waters into fresh, but rather that ecologically it was a permanent member of a freshwater fauna.

### Ecological Implications Based on the Osteology of *Pannoniasaurus*

The size of *Pannoniasaurus* makes it the largest known predator in the waters of this paleoenvironment [13]. Additionally, we consider the crocodile-like flattened skull (as indicated by the premaxilla and maxilla), to be a useful adaptation for water-level ambush hunting of terrestrial and shallow water prey.

It is difficult to estimate how the unknown girdle and limb elements of *Pannonisasaurus* may have looked. As far as we know, *Pannoniasaurus* had a primitive vertebral column, a posteriorly oriented ilium and an elongated humerus with a distal epiphysis, all most similar to aigialosaurs [3], [43]. These suggest that *P. inexpectatus* had an overall aigialosaur-like postcranial morphology (including plesiopelvia and plesiopedia). However, *Dallasaurus*, for example, has an anteriorly oriented, hydropelvic ilium in combination with primitive-looking proximal limb elements [2]; thus, a flattened, derived distal limb morphology is not impossible for that taxon as noted by Caldwell and Palci [4]. Nevertheless, one must be careful when trying to reconstruct unknown portions of these animals. For *Pannoniasaurus* a primitive morphology of the complete limbs in correlation with the primitive axial skeleton and pelvis is more probable, but far from certain. It is possible that the retention of a robust sacrum, pelvis and possibly non-paddle-like limbs were used to help to propel the body forward from the bottom during prey-capture in shallow water, similar to extant crocodiles.

### Phylogenetic Relationships of *Pannoniasaurus* and the Tethysaurinae

The phylogenetic relationships of mosasaurs and their kin have gone through radical changes in recent years, with no doubt a great deal more revision to come [11]. Aigialosaurs, poorly known semi-aquatic squamates found in Tethyan shallow marine sediments, possess a skull that is as typically mosasaur as are the skulls of any of the mosasaurines, halisaurines, tylosaurines and plioplatecarpines. However, the postcranium is much more similar to terrestrial lizards, even though there are still only a small number of taxa and specimens that are recognized as aigialosaurs [53]. In contrast, mosasaurs have been known for a very long time and have been the subject of numerous studies examining literally thousands of specimens housed in the museums of the world [6], [54], [32].

**Figure 10:**
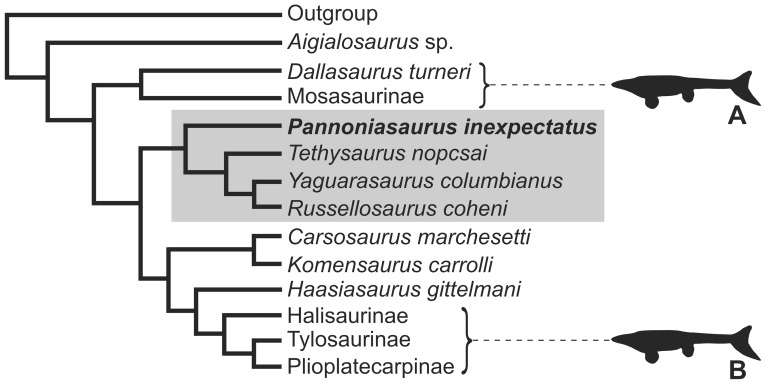
Convergent evolution of hydropelvia *sensu* Caldwell and Palci [**4**]. Dark grey box indicates the new aigialosaur clade Tethysaurinae. Black mosasaur images indicate the convergent evolution of mosasaur traits from two separate lineages of aigialosaurs: *Dallasaurus turneri* is the sistergroup to the mosasaurine “mosasaurs” (A), and *Haasiasaurus gittelmani* is the sistertaxon to halisaurine, tylosaurine and plioplatecarpine “mosasaurs” (B).

Our results (Figures 8, 9, and 10) clearly illustrate and support previous conclusions [2−4], that hydropelvia and hydropedia evolved at least twice within aigialosaur squamates. *Aigialosaurus* remained the sister taxon to all other mosasauroids, with *Dallasaurus* placed as the the sister group to the Mosasaurinae (Figure 9A) (Hydropelvia I, Figure 10A), while *Carsosaurus*, *Komensaurus*, and *Haasiasaurus* remained basal to Halisaurinae (Figure 9C) and Tylosaurinae+Plioplatecarpinae (Figure 9D) (Hydropelvia II; Figure 10B).

The principle impact of recovering *Pannoniasaurus* as a member of the Tethysaurinae is the topological shift of the entire clade such that it is the sister clade to the clade composed of (*Carsosaurus* (*Komensaurus* (*Haasiasaurus* (Halisaurinae (Tylosaurinae, Plioplatecarpinae))))). Russellosaurina *sensu* Polcyn and Bell [37] clearly requires revision as it was previously defined as “all mosasaurs more closely related to Tylosaurinae and Plioplatecarpinae, the genus *Tethysaurus*, their common ancestor and all descendants than to Mosasaurinae” [37].

Considering the primitive cranial and postcranial characters of *Tethysaurus* [33], *Russellosaurus* [37], *Yaguarasaurus* [38], [41] and *Pannoniasaurus*, which are typical of aigialosaurs, it is reasonable to regard these animals as large-bodied aigialosaurs. Although it is unfortunate that there is almost no postcranial material known for *Yaguarasaurus* and *Russellosaurus*, it seems likely that when such remains are recovered they will resemble those of *Tethysaurus* and *Pannoniasaurus*.

The distinctiveness of the clade Tethysaurinae amongst aigialosaurs suggests very strongly that future discoveries will identify even more lineages of basal aigialosaurs, some perhaps with even more specific affinities to the diverse assemblage of hydropelvic forms. Because in the near future the discovery of new taxa, and as a result new analyses and different phylogenies seem likely, the present paper does not address the problem of cleaning the nomenclature of mosasauroid clades, a problem already presented by previous authors [11].

### Previous Reports of Mosasauroids and Relatives from Freshwater Sediments

The Pythonomorpha (dolichosaurs, aigialosaurs and mosasaurs) are conventionally characterized as ecologically marine lizards. A marine ecology is ascribed to them primarily because nearly 100% of their fossil occurrences are from rocks that are interpreted as having been deposited in a wide variety of marine environments (e.g., inland seaways, patch reef lagoons, a variety of nearshore and foreshore environments). The geological time range of the Pythonomorpha currently is recognized as beginning in the Barremian (*Kaganaias* sp. in Japan [10]) and terminating at the end of the Maastrichtian [6]. Though there is little doubt that the deep ancestry of pythonomorphs finds a common ancestor with an as yet unidentified sister group amongst terrestrial lizards and snakes [55], it is not known if their subsequent evolution took place in coastal environments and they adapted directly to marine and freshwater environments, or if they evolved on land, primarily adapting to freshwater and later inhabiting the seas. Though pythonomorph fossils have been recognized as coming from marine sediments since Cuvier [54], only recently have a few discoveries [9], [10], [13], [39], [40] raised the possibility of a freshwater ecology for a few taxa.

The mosasaur *Plioplatecarpus* has been reported from freshwater sediments [9] from the Maastrichtian of Canada. It is not clear whether this report of a well-known marine genus demonstrates that some species of mosasaurs regularly exploited estuarine or freshwater environments, or if this specimen represents nothing more than a stochastic occurrence with no ecological implications whatsoever. The lack of similar finds suggests the latter.

*Goronyosaurus*, an unusual mosasaur from Nigeria and Niger is thought to have possibly exploited estuarine environments based on its skull morphology, but none of its remains are known from freshwater sediments [56], [57]. As there is no corroborating evidence to suggest anything other than a marine ecology for *Goronyosaurus*, we take a conservative view and consider it to be a marine mosasaur.

The recent publication [10] that reported *Kaganaias*, a long-bodied platynotan lizard from Lower Cretaceous freshwater sediments of Japan used the term mosasauroid loosely, as a collective term for dolichosaurs, pontosaurs, aigialosaurs, and mosasaurs. However, *Kaganaias*, as pointed out by the authors [10], is a primitive dolichosaur-like platynotan and might represent the first stages of aquatic adaptation among basal pythonomorphs.

These finds mentioned above were not clear evidence of mosasauroids adapted to freshwater environments. However, a large number of remains of a new species of mosasauroid (described here as *Pannoniasaurus*), originating from multiple individuals were collected during several excavations at the Iharkút locality after its discovery in 2000.

The first known specimen of *Pannoniasaurus* was the dorsal vertebra found on the waste dump of the Ajka coal mine in 1999, and more vertebrae were unearthed after the discovery of the Iharkút locality. However, these vertebrae were fragmentary and thus were first considered as belonging to large terrestrial varanoid lizards based on their characters such as precondylar constriction and oblique articulation of their centra [46]. Dorsal vertebrae of *Pannoniasaurus* strongly resemble varanoid vertebrae and terrestrial varanoids do occur in some Cretaceous localities (e.g. *Estesia mongoliensis* [58]). Thus, only after the discovery of more material (e.g. quadrates) was it recognized that these remains from Iharkút demonstrate the presence of a mosasauroid in the fauna. Later, other bones were identified in the material, and large-scale excavations at the locality continued, yielding more remains of *Pannoniasaurus,* and more evidence for the freshwater occurrence for multiple individuals of a basal mosasauroid [13], [39], [40,]. *Pannoniasaurus* became the first mosasauroid to be represented by multiple specimens from freshwater sediments. As a matter of fact, even if some pannoniasaurs were capable of moving back and forth from fluvial systems to the estuaries, or even further into marine environments, similar to extant La Plata dolphins but in contrast to exclusively riverine dolphin species (e.g. Amazon river dolphin) [12], it would not be a rejection of our hypothesis of *Pannoniasaurus* being the first mosasaur adapted to a freshwater environment.

### Conclusions

Until now, mosasauroids have been regarded as an exclusively marine group. However, with the discovery and description of *Pannoniasaurus*, mosasauroid evolution is now understood as also having involved important and unsuspected adaptations to freshwater ecosystems. These adaptations have taken place within the new subfamily, Tethysaurinae, the clade in which we reconstruct *Pannoniasaurus*.

Whether or not *Pannoniasaurus* was restricted to freshwater environments, or perhaps instead was a seasonal, opportunistic migrant and consumer in these habitats, remains uncertain. Sedimentological, taphonomical, morphological and geochemical evidences suggest the former. In association with the facies analysis and depositional environment interpretations, the collected evidence indicates that *Pannoniasaurus* is best interpreted as an inhabitant of freshwater ecosystems. Currently, among derived pythonomorphs, *Pannoniasaurus*, whether being an obligatory freshwater animal or a seasonal or opportunistic migrant, remains the first and only know river-dwelling member of the clade including aigialosaurs and mosasaurs.

The evidence we provide here makes it clear that similar to some lineages of cetaceans, mosasauroids quickly radiated into a variety of aquatic environments, with some groups reinvading available niches in freshwater habitats, and becoming highly specialized within those ecosystems.

## Supporting Information

Appendix S1 **Inventory numbers of referred specimens.**
(DOC)

Appendix S2 **List of characters used for phylogenetic analysis.**
(DOC)

Appendix S3 **Taxon-character matrix used for phylogenetic analysis.**
(TXT)
