# Supplementary material for: The First Freshwater Mosasauroid (Upper Cretaceous, Hungary) and a New Clade of Basal Mosasauroids
Source: PLoS One. 2012 Dec 19;7(12):e51781. doi: 10.1371/journal.pone.0051781 (PMC3526648; doi:10.1371/journal.pone.0051781)
Supplement: Appendix S1 — Inventory numbers of referred specimens. (DOC) [file pone.0051781.s001.doc]

**Appendix S1 Inventory numbers of referred specimens.**

MTM (Magyar Természettudományi Múzeum, Hungarian Natural History Museum) 2007.24.1. premaxilla fragment; MTM 2007.25.1. premaxilla (Fig. S4C); MTM 2007.26.1. maxilla fragment; MTM 2007.29.1. right maxilla (Fig. S4D); MTM 2007.33.1. maxilla fragment; MTM 2007.22.1. left postorbitofrontal; MTM 2007.28.1. left postorbitofrontal (Fig. S4E); MTM 2007.37.1. left dentary (Fig. S4G); MTM 2007.37.2. right dentary; MTM 2007.77.1. left dentary fragment; MTM 2011.41.1. left splenial (Fig. S4I); MTM V.01.37. right splenial fragment; MTM 2007.38.1. right splenial; MTM 2007.34.1. right angular fragment; MTM 2007.35.1. right angular; MTM 2007.36.1. right angular (Fig. S4K); MTM 2007.23.1. left coronoid (Fig. S4J); MTM V.01.49. left surangular; MTM 2007.30.1. right surangular (Fig. S4L); MTM 2007.39.1. right articular (Fig. S4M); MTM 2007.78.1. - MTM 2007.78.91. isolated teeth (91 pcs) (Fig. S4H); MTM Gyn/106. cervical vertebra; MTM Gyn/108. cervical vertebra; MTM Gyn/113. cervical vertebra; MTM Gyn/120. cervical vertebra; MTM Gyn/121. cervical vertebra; MTM V.2000.19. cervical vertebra; MTM V.01.149. cervical vertebra (Fig. S4N); MTM 2007.49.1. cervical vertebra; MTM 2007.52.1. cervical vertebra; MTM 2007.57.1. cervical vertebra fragment; MTM 2007.58.1. cervical vertebra; MTM 2007.66.1. cervical vertebra; MTM 2007.68.1. cervical vertebra; MTM 2007.70.1. cervical vertebra; MTM 2007.71.1. cervical vertebra fragment; MTM 2007.75.1. cervical vertebra fragment; MTM 2007.76.1. cervical vertebra; MTM 2007.79.1. cervical vertebra fragment; MTM 2007.93.1. cervical vertebra; MTM 2007.94.1. cervical vertebra fragment; MTM Gyn/109. dorsal vertebra; MTM Gyn/111. dorsal vertebra; MTM Gyn/114. dorsal vertebra; MTM Gyn/116. dorsal vertebra (pathologic); MTM Gyn/123. dorsal vertebra; MTM Gyn/124. dorsal vertebra; MTM Gyn/125. dorsal vertebra; MTM Gyn/126. dorsal vertebra; MTM V.2000.21. dorsal vertebra (from the Ajka Coal Fm.); MTM V.01.212. dorsal vertebra; MTM V.01.222. dorsal vertebra (Fig. S4O); MTM 2007.32.1. dorsal vertebra; MTM 2007.44.1. dorsal vertebra; MTM 2007.45.1. dorsal vertebra; MTM 2007.47.1. dorsal vertebra; MTM 2007.48.1. dorsal vertebra; MTM 2007.50.1. dorsal vertebra fragment; MTM 2007.51.1. dorsal vertebra fragment; MTM 2007.53.1. dorsal vertebra; MTM 2007.54.1. dorsal vertebra; MTM 2007.55.1. dorsal vertebra fragment; MTM 2007.56.1. dorsal vertebra fragment; MTM 2007.59.1. dorsal vertebra; MTM 2007.60.1. dorsal vertebra; MTM 2007.61.1. dorsal vertebra; MTM 2007.62.1. dorsal vertebra; MTM 2007.63.1. dorsal vertebra; MTM 2007.64.1. dorsal vertebra fragment; MTM 2007.65.1. dorsal vertebra; MTM 2007.67.1. dorsal vertebra; MTM 2007.69.1. dorsal vertebra; MTM 2007.72.1. dorsal vertebra; MTM 2007.73.1. dorsal vertebra fragment; MTM 2007.74.1. dorsal vertebra; MTM 2007.80.1. dorsal vertebra; MTM 2007.81.1. dorsal vertebra; MTM 2007.82.1. dorsal vertebra; MTM 2007.83.1. dorsal vertebra; MTM 2007.84.1. dorsal vertebra fragment; MTM 2007.106.1. dorsal vertebra; MTM 2007.85.1. 1st sacral vertebra fragment; MTM Gyn/122. 1st sacral vertebra (Fig. S4P); MTM 2007.86.1. 1st sacral vertebra; MTM Gyn/121. 2nd sacral vertebra (Fig. S4Q); MTM Gyn/104. caudal vertebra (Fig. S4R); MTM Gyn/105. caudal vertebra; MTM Gyn/118. caudal vertebrae (2 pcs); MTM Gyn/119. caudal vertebra; MTM V.01.173. caudal vertebra; MTM 2007.46.1. caudal vertebra; MTM 2007.95.1. caudal vertebra; MTM 2007.96.1. caudal vertebra; MTM 2007.97.1. caudal vertebra; MTM 2007.98.1. caudal vertebra; MTM 2007.99.1. caudal vertebra; MTM 2007.100.1. caudal vertebra; MTM 2007.101.1. caudal vertebra; MTM 2007.102.1. caudal vertebra; MTM 2007.103.1. caudal vertebra; MTM 2007.104.1. caudal vertebra; MTM 2007.105.1. caudal vertebra; MTM Gyn/112. vertebrae (5 pcs); MTM Gyn/115. vertebrae (14 pcs); MTM Gyn/117. vertebra fragments (2 pcs); MTM V.2000.17. vertebra fragment; MTM 2007.90.1.- MTM 2007.90.11. vertebra fragments (11 pcs); MTM 2007.107.1. vertebra fragment; MTM 2007.87.1. rib proximal fragment; MTM 2007.88.1. rib proximal fragment; MTM 2007.89.1. rib proximal fragment (Fig. S4S); MTM V.01.43. right ilium; MTM 2007.40.1. left ilium (Fig. S4V); MTM 2007.41.1. left ilium; MTM 2007.43.1. ilium fragment; MTM 2007.42.1. left humerus proximal tip(Fig. S4T); MTM 2011.42.1. right humerus distal tip (Fig. S4U).
