# Supplementary material for: The First Freshwater Mosasauroid (Upper Cretaceous, Hungary) and a New Clade of Basal Mosasauroids
Source: PLoS One. 2012 Dec 19;7(12):e51781. doi: 10.1371/journal.pone.0051781 (PMC3526648; doi:10.1371/journal.pone.0051781)
Supplement: Appendix S2 — List of characters used for phylogenetic analysis. (DOC) [file pone.0051781.s002.doc]

**Appendix S2 List of characters used for phylogenetic analysis.**

1. Premaxilla predental rostrum I: total lack of a bony rostrum (0), or presence of any predental rostrum (1). In lateral profile, the anterior end of the premaxilla either exhibits some bony anterior projection above the dental margin, or the bone recedes posterodorsally from the dental margin. The derived condition produces a relatively taller lateral profile with an obvious "bow" or "prow."

2. Premaxilla predental rostrum II: rostrum very short and obtuse (0), or distinctly protruding (1), or very large and inflated (2). In *Clidastes* a short, acute, protruding rostrum (state 1) produces a "V-shaped" dorsal profile and, as far as is known, is peculiar to that genus. An alternative condition, described as U-shaped, include those taxa whose rostral conditions span the whole range of states of characters 1 and 2. Hence, the descriptive character is abandoned in favor of a more informative structure-based series.

3. Premaxilla shape: bone broadly arcuate anteriorly (0), or relatively narrowly arcuate or acute anteriorly (1). In virtually all lizards the premaxilla is a very widely arcuate and lightly constructed element, and the base of the intenarial process is quite narrow as in *Opetiosaurus* (=*Aigialosaurus*). All other mosasaurids have the derived condition of a very narrowed premaxilla with the teeth forming a tight curve and the internarial process being proportionally wider.

4. Premaxilla internarial bar width: narrow, distinctly less than half of the maximum width of the rostrum in dorsal view (0), or wide, being barely narrower than the rostrum (1).

5. Premaxilla intenarial bar base shape: triangular (0), or rectangular (1). A vertical cross section through the junction of the internarial bar and the dentigerous rostrum produces an inverted triangle in most taxa. But in the derived state, this cross section is transversely rectangular because the broad ventral surface of the bar is planar.

6. Premaxilla internarial bar dorsal keel: absent (0), or present (1). In the derived condition a ridge rises above the level of a normally smoothly continuous transverse arch formed by the bones of the anterior muzzle. Because of the distinct difference of form, these structures may not be homologous in *Tylosaurus* and *Mosasaurus*-*Plotosaurus*.

7. Premaxilla intenarial bar venter: with entrance for the fifth cranial (facial) nerve close to rostrum (0), or far removed from rostrum (1). The conduit that marks the path of the fifth cranial nerve from the maxilla into the premaxilla is expressed as a ventrolateral foramen within the premaxillo-maxillary sutural surface at the junction of the intenarial bar and the dentigerous rostrum. The derived condition includes a long shallow groove on the ventral surface of the bar. Anteriorly the groove becomes a tunnel entering the bone at an extremely shallow angle, but disappearing below the surface at least 1 cm behind the rostrum.

8. Frontal shape: sides sinusoidal (0), or bone nearly triangular and sides relatively straight (1). In the derived condition, the area above the orbits is expanded and an isosceles triangle is formed by the rectilinear sides. In certain taxa a slight concavity is seen above the orbits, but anterior and posterior to this there is no indication of a sinusoidal or recurved edge.

9. Frontal width: modified character to reflect more accurately, the variation seen in frontal length-to-width ratios. Mosasauroid frontals can be separated into three groups that generally have a maximum length to maximum width ratio of about 2:1, a group which has a ratio of between 1.5:1 and 2:1, and a group which has a ratio being generally equal to or less than 1.5:l. Element broad and short (0), intermediate dimension (1), or long and narrow (2).

10. Frontal narial emargination: frontal not invaded by posterior end of nares (0), or distinct embayment present (1). In some mosasauroids, the posterior ends of the nares are concomitant with the anterior terminus of the frontal-prefrontal suture and, therefore, there is no marginal invasion of the frontal by the opening. However, in other mosasauroids this suture begins anterior and lateral to the posterior ends of the nares, causing a short emargination into the frontal.

11. Frontal midline dorsal keel: absent (0), or low, fairly inconspicuous (1), or high, thin, and well-developed (2).

12. Frontal ala shape: sharply accuminate (0), or more broadly pointed or rounded (1). In state 0, the anterolateral edge of the ala is smoothly concave, thus helping to form the sharply pointed and laterally oriented posterior corners. In some Natantia, the anterolateral edge of the ala may be concave, but the tip is not sharp and directed laterally.

13. Frontal olfactory canal embrasure: canal not embraced ventrally by descending processes (0), or canal almost or completely enclosed below (1). In state 1, very short descending processes from sides of the olfactory canal surround and almost, or totally, enclose the olfactory nerve.

14. Frontal posteroventral midline: tabular boss immediately anterior to the frontal-parietal suture absent (0), or present (1). A triangular boss with a flattened ventral surface at the posterior end of the olfactory canal is the derived condition.

15. Frontal-parietal suture: opposing surfaces with low interlocking ridges (0), or with overlapping flanges (1). Plesiomorphically, an oblique ridge on the anterior sutural surface of the parietal intercalates between a single median posterior and a single lateral posterior ridge from the frontal. In the derived condition, these ridges are protracted into strongly overlapping flanges. The dorsal trace of the suture can be quite complex with a portion of the parietal embraced by the posterior extension of these frontal flanges.

16. Frontal-parietal suture overlap orientation: suture with oblique median frontal and parietal ridges contributing to overlap (0), or with all three ridges almost horizontal (1). In state 0, the median ridge from the frontal and the single parietal ridge are oriented at a distinct angle to the upper skull surface while the outer, or lateral, frontal ridge appears to be nearly horizontal. In *Tylosaurus nepaeolicus* and *T. proriger* (state 1), the obliquity of the intercalating ridges is reclined almost to the horizontal, greatly extending the amount of lateral overlap.

17. Frontal invasion of parietal: lateral sutural flange of frontal posteriorly extended (0), or median frontal sutural flange posteriorly extended (1), or both extended (2). In all mosasaurines, the oblique median frontal sutural ridge extends onto the dorsal surface of the parietal table and embraces a portion of the anterior table within a tightly crescentic midline embayment. In *Plioplatecarpus*, and *Platecarpus*, the lateral oblique sutural ridge from the frontal is greatly protracted posteriorly to cause a large, anteriorly convex embayment in the dorsal frontal-parietal suture. In this case the entire posterolateral corners of the frontal are extended backward to embrace the anterolateral portions of the parietal table. Consequently, the parietal foramen is very widely embraced laterally and the oblique anterior sutural ridge of the parietal occupies a position inside the embayment within the frontal.

18. Frontal medial invasion of parietal II: if present, posteriorly extended median sutural ridge short (0), or long (1). The median oblique sutural ridge discussed in character 17 is either short, not reaching back to the parietal foramen (state 0), or tightly embraces the foramen while extending backward to a position even with or beyond its posterior edge (state 1).

19. Parietal length: dorsal surface relatively short with epaxial musculature insertion posterior between suspensorial rami only (0), or dorsal surface elongate with epaxial musculature insertion dorsal as well as posterior (1).

20. Parietal table shape: modified character to reflect variation if parietal table morphology in basal forms. Generally rectangular to trapezoidal with sides converging but not meeting (0), triangular with straight sides contacting in front of suspensorial rami (1), triangular table with posterior portion forming parasagittal crest or ridge (2).

21. Parietal foramen size: relatively small (0), or large (1). If the foramen is smaller than or equal to the area of the stapedial pit, it is considered small. If the foramen is significantly larger or if the distance across the foramen is more than half the distance between it and the nearest edge of the parietal table, the derived state is achieved.

22. Parietal foramen position I: foramen generally nearer to center of parietal table, well away from frontal-parietal suture (0), or close to or barely touching suture (1), or huge foramen straddling suture and deeply invading frontal (2). Generally in state 1, the distance from the foramen to the suture is about equal to or less than one foramen length.

23. Parietal foramen ventral opening: opening is level with main ventral surface (0), or opening surrounded by a rounded, elongate ridge (1).

24. Parietal posterior shelf: presence of a distinct horizontal shelf projecting posteriorly from between the suspensorial rami (0), or shelf absent (1). In some mosasauroids, a somewhat crescent-shaped shelf(in dorsal view) lies at the posterior end of the bone medial to and below the origination of the suspensorial rami.

25. Parietal suspensorial ramus compression: greatest width vertical or oblique (0), or greatest width horizontal (1). In *Tylosaurus*, the anterior edge of the ramus begins very low on the lateral wall of the descending process, leading to formation of a proximoventral sulcus, but the straps are horizontal distally.

26. Parietal union with supratemporal: suspensorial ramus from parietal overlaps supratemporal without interdigitation (0), or forked distal ramus sandwiches end of supratemporal (1).

27. Prefrontal supraorbital process: process absent, or present as a very small rounded knob (0), or a distinct to large, triangular or rounded, overhanging wing (1).

28. Prefrontal contact with postorbitofrontal: no contact at edge of frontal (0), or elements in contact there (1). State 1 is usually described as the frontal being emarginate above the orbits. Often this character can be evaluated by examining the ventral surface of the frontal where depressions outline the limits of the sutures for the two ventral elements.

29. Prefrontal-postorbitofrontal overlap: prefrontal overlapped ventrally by postorbitofrontal (0), or prefrontal overlapped laterally (1). Postorbitofrontal ventral overlap of the prefrontal is extreme in *Platecarpus tympaniticus*, and *Plioplatecarpus*, such that there is even a thin flange of the frontal interjected between the prefrontal above and the postorbitofrontal below. In *T. proriger*, the postorbitofrontal sends a long narrow process forward to fit into a lateral groove on the prefrontal. In *Plesiotylosaurus*, the overlap is relatively short and more oblique, and there is no groove on the prefrontal.

30. Postorbitofrontal shape: narrow (0), or wide (1). In *Clidastes* and *Globidensini*, the lateral extent of the element is almost equal to half the width of the frontal and the outline of the bone is basically squared, while in all other ingroup and outgroup taxa, it is a fairly narrow hourglass shape.

31. Postorbitofrontal transverse dorsal ridge: absent (0), or present (1). In state 1, an inconspicuous, low and narrowly rounded ridge traces from the anterolateral corner of the parietal suture across the top of the element to disappear behind the origin of the jugal process.

32. Postorbitofrontal squamosal ramus: does (0), or does not (1), reach end of supratemporal fenestra.

33. Maxilla tooth number: 20-24 (0), or 17-19 (1), or 15-16 (2), or 14 (3), or 13 (4), or 12 (5).

34. Maxillo-premaxillary suture posterior terminus: suture ends above a point that is anterior to or even with the midline of the fourth maxillary tooth (0), or between the fourth and ninth teeth (1), or even with or posterior to the ninth tooth (2). These somewhat arbitrary divisions of the character states are meant to describe in more concrete terms those sutures that terminate far anteriorly, those that terminate less anteriorly, and those that terminate near the midlength of the maxilla, respectively.

35. Maxilla posterodorsal process: recurved wing of maxilla dorsolaterally overlaps a portion of the anterior end of prefrontal (0), or process absent (1).

36. Maxilla posterodorsal extent: recurved wing of maxilla prevents emargination of prefrontal on dorsolateral edge of external naris (0), or does not (1).

37. Jugal posteroventral angle: angle very obtuse or curvilinear (0), or slightly obtuse, near 120 degrees (1), or 90 degrees (2).

38. Jugal posteroventral process: absent (0), or present (1).

39. Ectopterygoid contact with maxilla: present (0), or absent (1).

40. Pterygoid tooth row elevation: teeth arise from robust, transversely flattened, main shaft of pterygoid (0), or teeth arise from thin pronounced vertical ridge (1). Plesiomorphically, the teeth emanate from the relatively planar surface of the thick, slightly dorsoventrally compressed main shaft of the pterygoid. In the derived state a tall, thin dentigerous ridge emanates ventrally from a horizontal flange that forms the base of the quadratic ramus and the ectopterygoid process, thus causing the main shaft to be trough-shaped.

41. Pterygoid tooth size: anterior teeth significantly smaller than marginal teeth (0), or anterior teeth large, approaching size of marginal teeth (1).

42. Quadrate suprastapedial process length: process short, ends at a level well above midheight (0), or of moderate length, ending very near midheight (1), or long, ending distinctly below midheight (2).

43. Quadrate suprastapedial process constriction: distinct dorsal constriction (0), or virtually no dorsal constriction (1). Lack of constriction results in an essentially parallel-sided process in posterodorsal view, but can also include the tapering form characteristic of some *Tylosaurus*.

44. Quadrate suprastapedial ridge: if present, ridge on ventromedial edge of suprastapedial process indistinct, straight and/or narrow (0), or ridge wide, broadly rounded, and curving downward, especially above stapedial pit (1).

45. Quadrate suprastapedial process fusion: no fusion present (0), or process fused to elaborated process from below (1). A posterior rugose area may be inflated and broadened mediolaterally to partially enclose the ventral end of a broad and elongate suprastapedial process as in *Halisaurus*. In *Globidens*, *Prognathodon*, and *Plesiotylosaurus*, the process is fused ventrally to a narrow pedunculate medial extension of the tympanic rim. A similar condition is present in *Ectenosaurus*, except that the tympanic rim is not medially extended and has a short projection that overlaps a portion of the suprastapedial process posteriorly.

46. Quadrate stapedial pit shape: pit broadly oval to almost circular (0), or relatively narrowly oval (1), or extremely elongate with a constricted middle (2). In state 0, the length to width ratio is less than 1.8:1; in state 1, it ranges from 1.8:1 to 2.4:1; and in state 2, it is greater than 2.4:1.

47. Quadrate infrastapedial process: absent (0) (not figured), or present (1). The infrastapedial process can be expressed as an elaborated or swollen rugose area on the posteroventral face of the main quadrate shaft, as an extension of the ascending posteroventral portion of the tympanic rim, or as a small protuberance emanating from the ventral end of the anterior meatal wall. These various structures are probably not homologous.

48. Quadrate posteroventral ascending tympanic rim condition: small, low ridge present (0), or a high, elongate crest (1), or crest extremely produced laterally (2). In state 1, this extended rim causes a fairly deep sulcus in the ventral portion of the intratympanic cavity. In *Plioplatecarpus*, the entire lower tympanic rim and ala are expanded into a large conch (state 2) which tremendously increases the depth of the intratympanic cavity.

49. Quadrate ala thickness: ala thin (0), or thick (1). In state 0, the bone in the central area of the ala is only about 1 mm thick in medium-sized specimens and that area is usually crushed or completely destroyed. Alternatively, the ala extends from the main shaft with only minor thinning, providing a great deal of strength to the entire bone.

50. Quadrate conch: ala and main shaft encompassing a deeply bowled area (0), or alar concavity shallow (1). A relatively deeper sulcus in the anterior part of the intratympanic cavity and more definition between the ala and the main shaft are features of the plesiomorphic state.

51. Quadrate ala groove: absent (0), or long, distinct, and deep groove present in anterolateral edge of ala (1).

52. Quadrate median ridge: thin, high ridge, dorsal to ventral (0), or low and rounded with divergent ventral ridges (1).

53. Quadrate ventral condyle: condyle saddle-shaped, concave in anteroposterior view (0), or gently domed, convex in any view (1).

54. Quadrate anterior ventral condyle modification: no upward deflection of anterior edge of condyle (0), or distinct deflection present (1). A relatively narrow bump in the otherwise horizontal trace of the anterior articular edge is also supertended by a sulcus on the anteroventral face of the bone.

55. Basisphenoid pterygoid process shape: process relatively narrow with articular surface facing mostly anterolateraly (0), or somewhat thinner, more fan-shaped with a posterior extension of the articular surface causing a more lateral orientation (1).

56. Basioccipital tubera size: short (0), or long (1). Long tubera are typically parallel-sided in posterior profile and protrude ventrolaterally at exactly 45 degrees from horizontal. Short tubera have relatively large bases that taper distally, and emanate more horizontally.

57. Basioccipital tubera shape: tubera not anteroposteriorly elongate (0), or anteroposteriorly elongate with rugose ventrolateral surfaces (1).

58. Basioccipital canal: Modified to reflect variation seen in basilar artery entrance in floor of basioccipital. In *Yaguarasaurus*, *Platecarpus planifrons* and possibly *Angolasaurus*, the basilar arteries enter the basioccipital as a pair of foramen, separated by a median septum. In *Ectenosaurus*, *Platecarpus* and *Plioplatecarpus* a bilobate tunnel enters the basioccipital dorsally, passes forwards into the basisphenoid, and exits that bone dorsally and laterally to the braincase. No canal (0), a small pair separated by median septum (1), a large pair separated by median septum (2), a single bilobate canal (3).

59. Dentary tooth number I: 20-24 (0), or 17-19 (1), or 15-16 (2), or 14 (3), or 13 (4), or 12 (5), or <12 (6). It is easy to assume this character is correlated with the number of maxillary teeth, except that is not the case in *Ectenosaurus clidastoides*, which has 16 or 17 maxillary teeth and only 13 dentary teeth. Therefore, this character is included as different from character 35.

60. Dentary anterior projection: projection of bone anterior to first tooth present (0), or absent (1).

61. Dentary anterior projection length: short (0), or long (1). In the derived state, the projection of bone anterior to the first tooth is at least the length of a complete tooth space.

62. Dentary medial parapet: parapet positioned at base of tooth roots (0), or elevated and straplike, enclosing about half of height of tooth attachment in shallow channel (1), or strap equal in height to lateral wall of bone (2). The two derived states are possibly sequential stages of modification from a classically pleurodont dentition to the typical mosasaur "subthecodont" dentition.

63. Splenial-angular articulation shape: splenial articulation in posterior view almost circular (0), or laterally compressed (1), or intermediate (2).

64. Splenial-angular articular surface: essentially smooth concavo-convex surfaces (0), or distinct horizontal tongues and grooves present (1). Angulars of many *P. tympaniticus* have three or four arcuate ridges that fit into grooves in the contacting surface of the splenial. Thus, the splenial rotated horizontally on the angular about a vertical line. In *Plioplatecarpus*, the tongues and grooves are longer and deeper, thinner, and more numerous, a condition that absolutely restricts the anterior mandible to mediolateral movement in a horizontal plane.

65. Coronoid shape: coronoid with slight dorsal curvature, posterior wing not widely fan-shaped (0), or very concave above, posterior wing greatly expanded (1).

66. Coronoid posteromedial process: small but present (0), or absent (1).

67. Coronoid medial wing: does not reach angular (0), or contacts angular (1).

68. Coronoid posterior wing: without medial crescentric pit (0), or with distinct excavation (1). In state 1, there is a posteriorly open, C-shaped excavation in the medial side of the posterior wing of this element.

69. Surangular coronoid buttress: low, thick, about parallel to lower edge of mandible (0), or high, thin, rapidly rising anteriorly (1). A rounded dorsal edge of the surangular remains almost parallel to the ventral edge as it approaches the posterior end of the coronoid, meeting the latter element near its posteroventral edge in state 0. In the derived condition, the dorsal edge rises and thins anteriorly until meeting the posterior edge of the coronoid near its apex, producing a triangular posterior mandible in lateral aspect.

70. Surangular-articular suture position: behind condyle in lateral view (0), or at middle of glenoid on lateral edge (1). In the derived condition, there is usually an interdigitation in the dorsal part of the suture.

71. Surangular-articular lateral suture trace: suture descends and angles or curves anteriorly (0), or is virtually straight throughout its length (1). In state 1, the suture trails from the glenoid posteriorly about halfway along the dorsolateral margin of the retroarticular process, then abruptly turns anteriorly off the edge and strikes in a straight line for the posterior end of the angular.

72. Articular retroarticular process inflection: moderate inflection, less than 60 degrees (0), or extreme inflection, almost 90 degrees (1). In *Mosasaurus*, *Plotosaurus*, and *Prognathodon overtoni* the posterior terminus of the lower jaw lies almost horizontal, probably allowing for more muscle attachment.

73. Articular retroarticular process innervation foramina: no large foramina on lateral face of retroarticular process (0), or one to three large foramina present (1).

74. Tooth surface I: teeth finely striate medially (0), or not medially striate (1). In "Russellosaurinae" medial tooth striations are very fine and groups of tightly spaced striae are usually set apart by facets, leading to a fasciculate appearance.

75. Tooth surface II: teeth not coarsely textured (0), or very coarsely ornamented with bumps and ridges (1). In both species of *Globidens* and in *Prognathodon overtoni*, the coarse surface texture is extreme, consisting of thick pustules, and vermiform or anastomosing ridges.

76. Tooth facets: absent (0), or present (1). *Halisaurus* teeth are smoothly rounded except for the inconspicuous carinae. *Clidastes* is described in numerous places as having smooth unfaceted teeth, but many immature individuals and some larger specimens have teeth with three distinct facets on the medial faces. Adult *Tylosaurus proriger* has indistinct facets. *Mosasaurus* has taken this characteristic to the extreme.

77. Tooth fluting: absent (0), or present (1). In *Ectenosaurus*, and some *Platecarpus planifrons*, several broadly rounded vertical ridges alternate with shallow, round-bottomed grooves completely around the teeth.

78. Tooth inflation: crowns of posterior marginal teeth conical, tapering throughout (0), or crowns of posterior marginal teeth swollen near the tip or above the base (1). The rear teeth of *Globidens* and *Prognathodon overtoni* are distinctly fatter than other mosasauroid teeth, but those of *P. rapax* are also swollen immediately distal to the base.

79. Tooth carinae I: absent (0), or present but extremely weak (1), or strong and elevated (2). *Halisaurus* exhibits the minimal expression of this character (state 1) in that its marginal teeth are almost perfectly round in cross section; the carinae are extremely thin and barely stand above the suface of the teeth. *Globidens* is convergent in the strict sense of the character, but this is probably a result of obliteration of the carinae by extreme inflation.

80. Tooth carinae serration: absent (0), or present (1).

81. Tooth replacement mode: replacement teeth form in shallow excavations (0), or in subdental crypts (1). All mosasauroids that can be evaluated have an "anguimorph" type of tooth replacement, which is to have interdental positioning of replacement teeth and resorption pits associated with each. In some taxa, the resorption pits remained shallow, but in others, the pits deeply invaded the bony bases of the functional teeth and replacement teeth developed mostly in chambers underneath them. Such a change may be a necessary consequence of the "subthecodont" type of tooth implantation.

82. Atlas neural arch: notch in anterior border (0), or no notch in anterior border (1).

83. Atlas synapophysis: extremely reduced (0), or large and elongate (1). In state 1, a robust synapophysis extends well posteroventral to the medial articular surface for the atlas centrum, and it may be pedunculate (*Clidastes*) or with a ventral "skirt" that gives it a triangular shape (*Mosasaurus*). A very small triangular synapophysis barely, if at all, extends posterior to the medial articular edge in state 0.

84. Zygosphenes and zygantra: absent (0), or present (1). This character assesses only the presence of zygosphenes and zygantra, not their relative development. Nonfunctional and functional are considered as present.

85. Zygosphene and zygantra number: present on many vertebrae (0), or present on only a few (1).

86. Hypapophyses: last hypapophysis occurs on or anterior to seventh vertebra (0), or on ninth or tenth vertebra (1). State 1 is reported in *Mosasaurus missouriensis* (31), but this author noted that the last hypapophysis occurred on the seventh cervical.

87. Synapophysis height: facets for rib articulations tall and narrow on posterior cervicals and anterior trunk vertebrae (0), or facets ovoid, shorter than the centrum height on those vertebrae (1).

88. Synapophysis length: synapophyses of middle trunk vertebrae not laterally elongate (0), or distinctly laterally elongate (1). The lateral extension of the synapophyses from the middle of the trunk is as much as 70-80% of the length of the same vertebra in the derived state.

89. Synapophysis ventral extension: synapophyses extend barely or not at all below ventral margin of cervical centra (0), or some extend far below ventral margin of centrum (1). In the derived state, two or more anterior cervical vertebrae have rib articulations that dip well below the centrum, causing a very deeply concave ventral margin in anterior profile.

90. Zygapophysis development: zygapophyses present far posteriorly on trunk vertebrae (0), or zygapophyses confined to anterior trunk series (1). Plesiomorphically, zygapophyses extend at least to the sacral area.

91. Vertebral condyle inclination: condyles of trunk vertebrae inclined (0), or condyles vertical (1).

92. Vertebral condyle shape I: condyles of anteriormost trunk vertebrae extremely dorsoventrally depressed (0), or slightly depressed (1), or essentially equidimensional (2). In state 0, posterior height:width ratios of anterior trunk vertebrae are close to 2:1. In state 1, they are close to 4:3, but posterior to this the ratio decreases as the vertebrae become proportionally higher.

93. Vertebral condyle shape II: condyles of posterior trunk vertebrae not higher than wide (0), or slightly compressed (1). In the derived condition, the posterior condylar aspect reveals outlines that appear to be higher than wide and even perhaps slightly subrectangular, due to the slight emargination for the dorsal nerve cord.

94. Vertebral synapophysis dorsal ridge: sharp ridge absent on posterior trunk synapophyses (0), or with a sharp-edged and anteriorly precipitous ridge connecting distal synapophysis with prezygapophysis (1). In the plesiomorphic condition, the ridge in question, if present, may be incomplete or it may be rounded across the crest with the anterior and posterior sides about equally sloping.

95. Vertebral length proportions: cervical vertebrae distinctly shorter than longest vertebrae (0), or almost equal or are the longest (1).

96. Presacral vertebrae number I: relatively few, 32 or less (0), or numerous, 39 or more (1). Here presacral vertebrae are considered to be all those anterior to the first bearing an elongate transverse process.

97. Presacral vertebrae number II: if few, then 28 or 29 (0), or 30 or 31 (1).

98. Sacral vertebrae number: two (0), or less than two (1). Numerous well-preserved specimens of derived mosasauroids have failed to show any direct contact of the pelvic girdle with vertebrae in the sacral area. Certainly, no transverse processes bear any type of concave facet for the ilium, and so it is generally assumed that a ligamentous contact was established with only one transverse process. Depending on one's perspective, it could be said that derived mosasauroids have either no or one sacral vertebra.

99. Caudal dorsal expansion: neural spines of tail all uniformly shortened posteriorly (0), or several spines dorsally elongated behind middle of tail (1).

100. Hemal arch length: hemal arches about equal in length to neural arch of the same vertebra (0), or length about 1.5 times greater than neural arch length (1). This ratio may be as great as 1.2:1 in state 0. Comparison is most accurate in the middle of the tail and is consistent even on those vertebrae in which the neural spines are also elongated.

101. Hemal arch articulation: arches articulating (0), or arches fused to centra (1). Among the outgroups, the hemal arches fuse to the centra generally only in the latest life stages. These are considered to have state 0 of this character. All mosasaurines have fused hemals and all "russellosaurines" have fused hemals. No mosasaurian is known to change this condition in any ontogenetic stage represented by fossil material, but the possibility that some very old "russellosaurines" fused the hemals awaits discovery.

102. Tail curvature: no structural downturn of tail (0), or tail with decurved posterior portion (1).

103. Body proportions: head and trunk shorter than or about equal to tail length (0), or head and trunk longer than tail (1).

104. Scapula/coracoid size: both bones about equal (0), or scapula about half the size of coracoid (1).

105. Scapula width: no anteroposterior widening (0), or distinct fan-shaped widening (1), or extreme widening (2). In the plesiomorphic condition, the anterior and posterior edges of the scapula encompass less than one quarter of the arc of a circle, but in state 1, the arc is increased to approximately one third. In state 2, the distal margin encompasses almost a half-circle and the anterior and posterior borders are of almost equal length.

106. Scapula dorsal convexity: if scapula widened, dorsal margin very convex (0), or broadly convex (1). In state 0, the anteroposterior dimension is almost the same as the proximodistal dimension. In state 1, the anteroposterior dimension is much larger.

107. Scapula posterior emargination: posterior border of bone gently concave (0), or deeply concave (1). In the derived condition, there is a deeply arcuate emargination on the posterior scapular border just dorsal to the glenoid. It is immediately bounded dorsally by a corner which begins a straight-edged segment that continues to the dorsal margin.

108. Scapula-coracoid fusion: ontogenetic fusion occurs (0), or no fusion at any life stage (1). Fully grown representatives of the outgroups and the only known specimen of *Opetiosaurus* (=*Aigialosaurus*) have fused the scapula and coracoid. This occurs in no other mosasauroid specimen, regardless of size.

109. Scapula-coracoid suture: unfused scapula-coracoid contact has interdigitate suture anteriorly (0), or apposing surfaces without interdigitation (1). Although all outgroup taxa fuse this suture ontogenetically, an interdigitate suture is present early in life; therefore, the interdigitate suture is assigned as the plesiomorphic state.

110. Coracoid neck elongation: neck rapidly tapering from medial corners to a relatively broad base (0), or neck gradually tapering to a relatively narrow base (1). The derived state of this character gives an outline of the bone which is nearly symmetrically and gracefully fan-shaped with gently concave, nearly equidistant sides.

111. Coracoid anterior emargination: present (0), or absent (1).

112. Humerus length: humerus distinctly elongate, about three or more times longer than distal width (0), or greatly shortened, about 1.5 to 2 times longer than distal width (1), or length and distal width virtually equal (2), or distal width slightly greater than length (3).

113. Humerus postglenoid process: absent or very small (0), or distinctly enlarged (1).

114. Humerus glenoid condyle: if present, condyle gently domed and elongate ovoid in proximal view (0), or condyle saddle-shaped, subtriangular in proximal view, and depressed (1), or condyle highly domed or protuberant and short ovoid to almost round in proximal view (2). In some taxa, the condylar surfaces of the limbs were finished in thick cartilage and there was no bony surface of the condyle to be preserved. This condition is scored as not represented. In some taxa, the glenoid condyle extends more proximally than does the postglenoid process (state 2) and it is not as ovoid as in the plesiomorphic state.

115. Humerus deltopectoral crest: crest undivided (0), or split into two separate insertional areas (1). In the derived state, the deltoid crest occupies an anterolateral or anterior position confluent with the glenoid condyle, while the pectoral crest occupies a medial or anteromedial area that may or may not be confluent with the glenoid condyle. The deltoid crest is often quite short, broad, and indistinct, being easily erased by degradational taphonomic processes.

116. Humerus pectoral crest: located anteriorly (0), or medially (1). In the derived condition, the pectoral crest is located near the middle of the flexor (or medial) side on the proximal end of the bone.

117. Humerus ectepicondylar groove: groove or foramen present on distolateral edge (0), or absent (1).

118. Humerus ectepicondyle: absent (0), or present as a prominence (1). A radial tuberosity is reduced in size in *Prognathodon*, but very elongated in *Plesiotylosaurus*.

119. Humerus entepicondyle: absent (0), or present as a prominence (1). The ulnar tuberosity protrudes posteriorly and medially from the posterodistal corner of the bone immediately proximal to the ulnar facet, causing a substantial dilation of the posterodistal corner of the humerus.

120. Radius shape I: radius not expanded anterodistally (0), or slightly expanded (1), or broadly expanded (2).

121. Ulna contact with centrale: broad ulnare prevents contact (0), or ulna contacts centrale (1). The derived condition causes the ulnare to be omitted from the border of the antebrachial foramen. There is usually a well-developed faceted articulation between the ulna and the centrale (or intermedium).

122. Radiale size: large and broad (0), or small to absent (1).

123. Carpal reduction: carpals number six or more (0), or five or less (1).

124. Pisiform: present (0), or absent (1).

125. Metacarpal I expansion: spindle-shaped, elongate (0), or broadly expanded (l). The broad expansion is also associated with an anteroproximal overhanging crest in every case observed.

126. Phalanx shape: phalanges elongate, spindle-shaped (0), or blocky, hourglass-shaped (1). All the basic taxa of *Mosasaurus* and *Plotosaurus* have phalanges that are slightly compressed and anteroposteriorly expanded on both ends.

127. Ilium crest: crest blade-like, points posterodorsally (0), or elongate, cylindrical (1).

128. Ilium acetabular area: arcuate ridge supertending acetabulum (0), or acetabulum set into broad, short V-shaped notch (1). The primitive ilium has the acetabulum impressed into the lateral wall of the bone, with a low narrow crest anterodorsally as the only surrounding topographic feature. In the derived condition, the acetabular area is set into a short, broadly V-shaped depression that tapers dorsally. The lateral walls of the ilium are therefore distinctly higher than the rim of the acetabulum.

129. Pubic tubercle condition: tubercle an elongate protuberance located closer to midlength of shaft (0), or a thin semicircular crest-like blade located close to acetabulum (1). In *Tylosaurus proriger*, this was scored as missing data because the structure is apparently lost.

130. Ischiadic tubercle size: elongate (0), or short (1). In the ancestral condition the tubercle is as long as the shaft of the ischium is wide, but it is only a short narrow spur in the derived state.

131. Astragalus: notched emargination for crural foramen, without pedunculate fibular articulation (0), or without notch, pedunculate fibular articulation present (1). For state 0, the tibia and fibula are of equal length about the crural foramen and the astragalus contacts both to about the same degree. The form of the latter element is symmetrical and subcircular with a sharp proximal notch. In state l, the outline of the element is basically reniform and the tibial articulation is on the same line as the crural emargination. The fibula is also shortened and its contact with the astragalus is narrow.

132. Appendicular epiphyses: formed from ossified cartilage (0), or from thick unossified cartilage (1), or epiphyses missing or extremely thin (2). Ends of limb bones show distinct vascularization and rugose surfaces indicating an apparently thick nonvascularized, unossified cartilage cap. Extremely smooth articular surfaces suggest the epiphyses were excessively thin or pehaps even lost.

133. Hyperphalangy: absent (0), or present (1).

134. Exit for basilar artery from basioccipital: reflects variation seen in exit for basilar artery from basioccipital: absent (0); through two ventral foramina (1); through single ventral foramina (2); through two foramina on anterodorsal basisphenoid (3).

135. Posterior thoracic vertebra: reflects variation in relative length seen in posterior thoracic vertebrae: posterior thoracic vertebra not markedly longer than anterior thoracics (0); markedly longer(1).
